# A Simple and Low-Cost Monitoring System to Investigate Environmental Conditions in a Biological Research Laboratory

**DOI:** 10.1371/journal.pone.0147140

**Published:** 2016-01-15

**Authors:** Akshay Gurdita, Heather Vovko, Mark Ungrin

**Affiliations:** Department of Comparative Biology and Experimental Medicine, Faculty of Veterinary Medicine, University of Calgary, Calgary, Alberta, Canada; Imperial College London, UNITED KINGDOM

## Abstract

Basic equipment such as incubation and refrigeration systems plays a critical role in nearly all aspects of the traditional biological research laboratory. Their proper functioning is therefore essential to ensure reliable and repeatable experimental results. Despite this fact, in many academic laboratories little attention is paid to validating and monitoring their function, primarily due to the cost and/or technical complexity of available commercial solutions. We have therefore developed a simple and low-cost monitoring system that combines a “Raspberry Pi” single-board computer with USB-connected sensor interfaces to track and log parameters such as temperature and pressure, and send email alert messages as appropriate. The system is controlled by open-source software, and we have also generated scripts to automate software setup so that no background in programming is required to install and use it. We have applied it to investigate the behaviour of our own equipment, and present here the results along with the details of the monitoring system used to obtain them.

## Introduction

Reproducibility is of central importance to rigorous scientific research, an issue that has garnered a great deal of attention recently across the scientific community [1–5]. While there is a strong tradition of monitoring environmental variables in clinical and industrial laboratories (often mandated by government regulation), in many academic settings equipment may simply be installed, and thereafter assumed to work to the manufacturer’s specifications. Laboratory monitoring systems can provide a range of critical quality-control parameters that might otherwise be overlooked. It has long been known that even relatively minor differences in temperature can significantly impact the growth of eukaryotic [6,7] and prokaryotic [8] cells and whole organisms [9–11], while the application of more recent technologies such as thermally-responsive surfaces can be disrupted by even brief changes in environment [12]. Manufacturer’s specifications cannot be relied upon to indicate true performance without further verification [13]. Even within normally-functioning systems, temperature excursions can occur in incubators or cold-storage systems while accessing samples, supply-line pressures can vary in CO2 or N2 gas lines, and containers can be incompletely or improperly closed.

Traditionally, monitoring systems to log this type of data have been restricted to critical systems in larger facilities due to cost. We have taken advantage of the ongoing drop in hardware prices to generate a system suitable for much broader application, monitoring individual pieces of equipment, and even specific experiments.

We present here the use of a low-cost single-board computer, coupled with a USB interface kit, to construct a cheap and effective laboratory monitoring system capable of logging a wide range of sensor inputs, presenting the data graphically via a web interface, and notifying the operator by email when parameters vary beyond the normal range. The system may be controlled from any web-enabled device, such as a personal computer, tablet or smart phone.

Built around the Raspberry Pi (RPi) [14–16], a widely available [17] low-cost linux-based single board general purpose computer, the system presented here is controlled via software written in Python; however, both sensors and the RPi are compatible with other programming languages. The operating system chosen for the RPi is Raspbian [18], a variant of the widely used Debian Linux [19]. Operating on a single standard-format USB power supply, the system is also therefore compatible with a wide range of external power-storage systems to allow for operation in remote locations or where electrical supply is unreliable.

## Materials and Methods

### Control computer

The core of the system is a Raspberry Pi 2 Model B (cat# 38Y6469, case cat# 68X2068, Newark element 14, Toronto, Ontario), although the software and sensors have also been tested successfully with the older Raspberry Pi Model B. A USB keyboard and HDMI monitor are required to set up the system, however once this process is complete these items are no longer required as the system can be operated remotely. The system was powered via a 5V 2A micro-USB B power supply (cat# 68X2071, Newark element 14, Toronto, Ontario). In some cases, backup power was provided by connecting an external battery pack (Patriot Fuel 9000mAh Tablet and Smartphone Mobile Rechargeable Battery, cat# PCPB90002, Amazon.ca) in between the power supply and the Raspberry Pi.

### Sensor hardware

Sensor systems were obtained from Phidgets.com (Calgary, Alberta). Sensor interaction is via a PhidgetInterfaceKit 8/8/8 (cat# 1018) for temperature and pressure sensors and digital inputs, or a PhidgetTemperatureSensor 4 (cat# 1048) for thermocouples. The individual sensors used were the Precision Temperature Sensor (cat# 1124), TPK-01 Bead Probe K-Type Thermocouple (cat# 3107), Absolute Air Pressure Sensor 20–400 kPa (cat# 1140), Absolute Air Pressure Sensor 15–115 kPa (cat# 1141), and Magnetic Contact Switch BR-1014 (cat# 3560).

### Sensor protection

In order to protect the thermocouple from damage it was encapsulated in ~1 mL of Sylgard 184 silicone (Dow Corning). This is not anticipated to have any effect on temperature readings at equilibrium, but may introduce a small lag if temperatures are changing rapidly. The shape and thickness of this coating may be adjusted by the user. The #1124 Precision Temperature Sensor is also suitable for encapsulation in silicone, which was used in some cases in order to permit use within a mammalian cell culture incubator, including sterilization with ethanol or peroxide without damaging the electronics. For this application a 3D printed mould allowing the sensor to replace a rubber stopper in the incubator’s access port was generated using a consumer-grade 3D printer (MakerBot Replicator 2, Thorstad Computing, Outlook Saskatchewan). The sensor was placed into this mould, and encapsulated in Sylgard 184 silicone. More details including the 3D printable file are included in S1 File.

### Set up and installation

For detailed instructions, see S1 Appendix. Briefly, the Raspberry Pi is initially booted as per the manufacturer’s instructions. A setup script may then be downloaded from the GitHub file sharing service, which automates the remainder of the process. This script installs the control software, as well as the necessary drivers for the Phidget sensors, a web server (“Lighttpd”) and a PHP graphics library (“pChart”) to enable graphical presentation of the sensor readouts. The source code for the control software is available via GitHub, and may be freely modified and extended to include additional sensor options as needed.

A table of parts and suppliers is included as S1 Table for convenience.

## Results and Discussion

**Fig 1:**
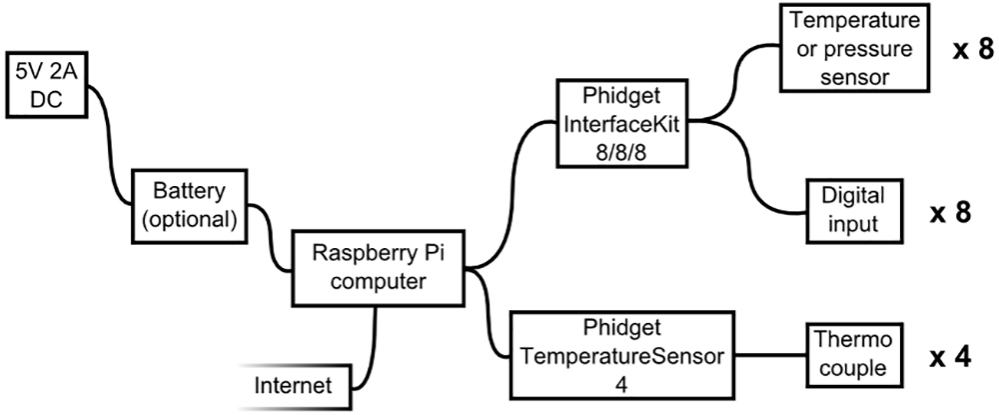
Monitoring system schematic. The Raspberry Pi is connected to an external power supply, with battery backup if desired, and to the internet to allow configuration, display instrument readings and send email alerts. It may be connected to an InterfaceKit and / or a thermocouple interface, which in turn can be connected to up to 8 pressure or temperature sensors, 8 digital inputs, and 4 thermocouples respectively.

The monitoring system is installed via an automated script, and operated via a web interface. The underlying software is fully open-sourced, and is accessible to the user for customization when logging into the RPi either directly, or remotely via the SSH protocol (see S1 Appendix). The architecture of the system is diagrammed in Fig 1.

**Fig 2:**
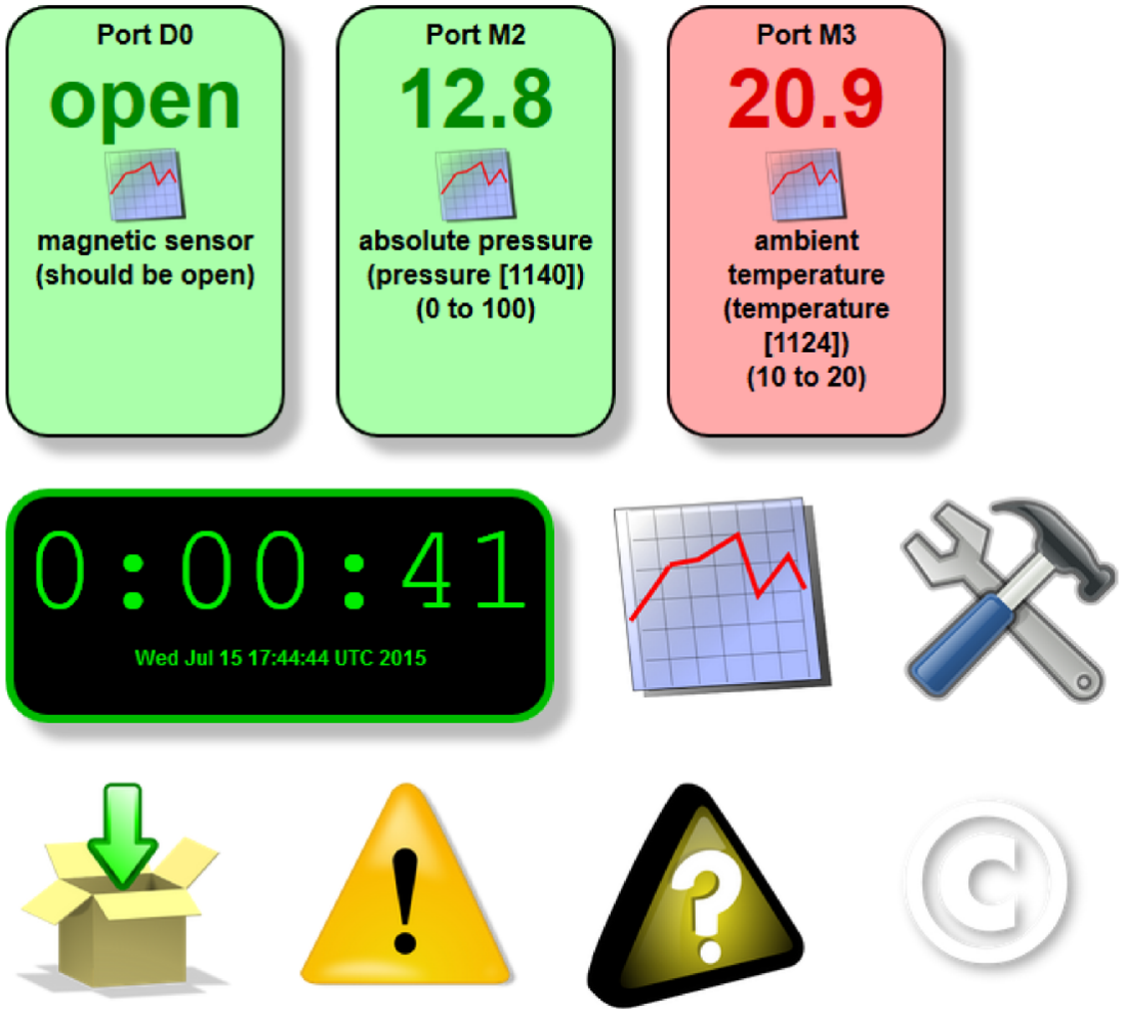
Real-time sensor monitoring. The current sensor status can be accessed using a web browser on any web-enabled device. Sensors whose output falls inside a user-designated range are shown in green, while sensors with values outside this range are shown in red. Icons are links to plot recorded data, change settings, download data, see alert log, check debug messages and view GPL copyright notice.

The software provided here (see Supporting Information) supports temperature and pressure sensing, including via broad temperature range thermocouple probes, as well as digital / binary inputs including contact and proximity sensors, or direct connection to alarm indicators present on some laboratory equipment. The sensors are read periodically (by default, every minute), and the readings are then stored in log files that may be accessed via a web server on the RPi. The RPi then processes this data to provide a real-time visual indication of system status (Fig 2) and graphs of sensor data over time, and send email alerts.

In order to validate sensor readings, data from the #3107 K-type thermocouple was recorded in both an ice-water bath and a boiling bath, and from a #1124 temperature sensor in the ice-water bath. The manufacturer’s listed error for both sensors is ± 0.75°C. In the ice-water bath (273.15 K) the thermocouple reported 273.07 ± 0.026 K; while the 1124 sensor reported 274.03 ± 0.0 (temperature variability was less than the 0.22 degree temperature resolution of this detector). Both represent 45 readings taken over a period of 23 minutes.

Our laboratory is located in Calgary, Canada, at sufficient altitude (~1,100 m) to affect the boiling point of water. With a nominal pressure of 88.7 kPa at this altitude, we obtained 26 readings over 13 minutes from the #1140 pressure sensor, which indicated a pressure of 88.54 kPa for all readings (variability was less than the device resolution of 413 Pa)

Using the Clausius-Clapeyron equation with this measured atmospheric pressure of 88.54 kPa, the expected boiling point of water is 369.33 K. The results from the #3107 thermocouple immersed in boiling water were 369.19 ± 0.53 K, from 87 readings taken over the course of 43 minutes.

In combination these results give us confidence that the thermocouple, temperature and pressure sensors are operating correctly.

**Fig 3:**
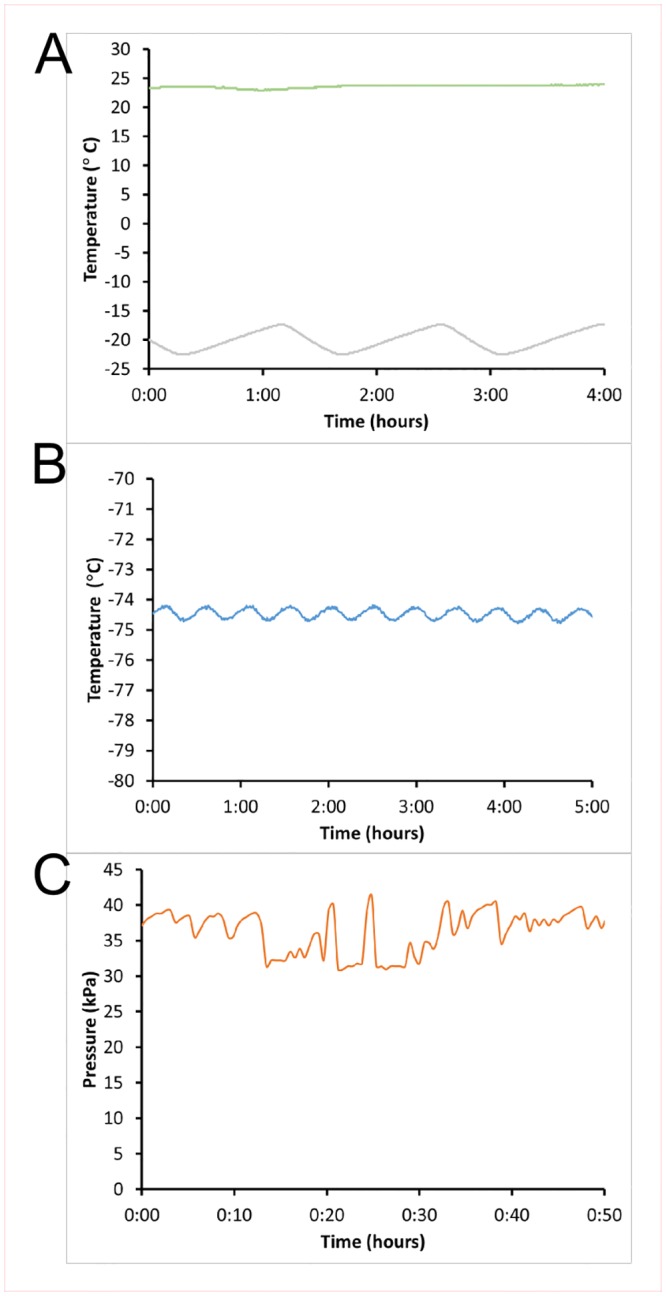
Representative basic laboratory monitoring results. A #1124 Temperature Sensor was placed in the top shelf of a laboratory freezer set to -20°C while a second such sensor was placed on a benchtop for room temperature monitoring (**A**). A #3107 K-type thermocouple was inserted via an external access port located on the top face of an upright ultra-low temperature freezer set to -80°C (**B**). A #1140 pressure sensor was used to monitor the pressure of carbon dioxide within a supply line. A second sensor was used to monitor the ambient room pressure, and the difference between the two plotted (**C**).

Subsequently the system was used to monitor various sensor readings in our laboratory. We first assessed temperature stability in a -20°C freezer, along with lab room temperature as a reference (Fig 3A). We were interested to note that the internal temperature underwent a regular cycle, varying through more than 5°C with a periodicity of just under 90 minutes. As this is a relatively new piece of equipment (~2 years old), it would not be surprising to find more dramatic excursions in older equipment, with potential impacts on sample and reagent preservation.

We then examined temperature stability in an ultra-low freezer, with a set point of -80°C via a K-type thermocouple (Fig 3B). While temperature stability was much better in this freezer, varying only on the order of one degree, it is interesting to note that the temperature reading is several degrees warmer than the set point, possibly due to the location of the thermocouple at the top of the freezer. Also, while thermocouple readings agreed closely with predicted values in freezing and boiling baths, the potential for increased deviations outside this range cannot be ruled out. Once again, this is a relatively new piece of equipment and we expect greater departures from the theoretical temperature are likely to occur in older systems. These freezers are commonly used to store high-value samples and reagents, but due to noise and heat-elimination issues are often installed in spaces separate from the main laboratory. As a consequence, purely local audible alarms to indicate failure conditions are often inadequate. Loss of temperature control may not be immediately detected by the user, particularly if it happens at night or during the weekend, making automated monitoring an important asset. While rated to -50°C due to concern about cracking of electrical wire insulation when flexed, under static conditions the #3107 thermocouple is suited to much lower temperatures and can read temperatures as low as -140°C or below.

We then examined the gas pressure in the supply lines delivering CO_2_ to our laboratory (Fig 3C). The fluctuations in pressure reflect CO_2_ use by three cell culture incubators connected to the same supply line in an adjacent room. The prominent peaks in the centre of the plot occurred when an incubator door was opened (halting CO_2_ injection) followed by a period of reduced pressure as the incubator injected additional CO_2_ to compensate for losses when the door was open. This data will allow rapid detection of gas supply failure conditions, including breaks in the supply line and / or tank exhaustion. With the accumulation of long-term data trends in gas consumption may also become apparent, giving warning of smaller leaks should they arise. NB it is important when connecting gas-line pressure sensors that the connection be robust and leak-proof, so as not to introduce a point of failure. When dealing with dangerous gases (or large volumes of inert gases that may become dangerous due to e.g. oxygen displacement) it is desirable to consult with a health and safety officer to ensure proper installation and adequate safeguards are in place.

**Fig 4:**
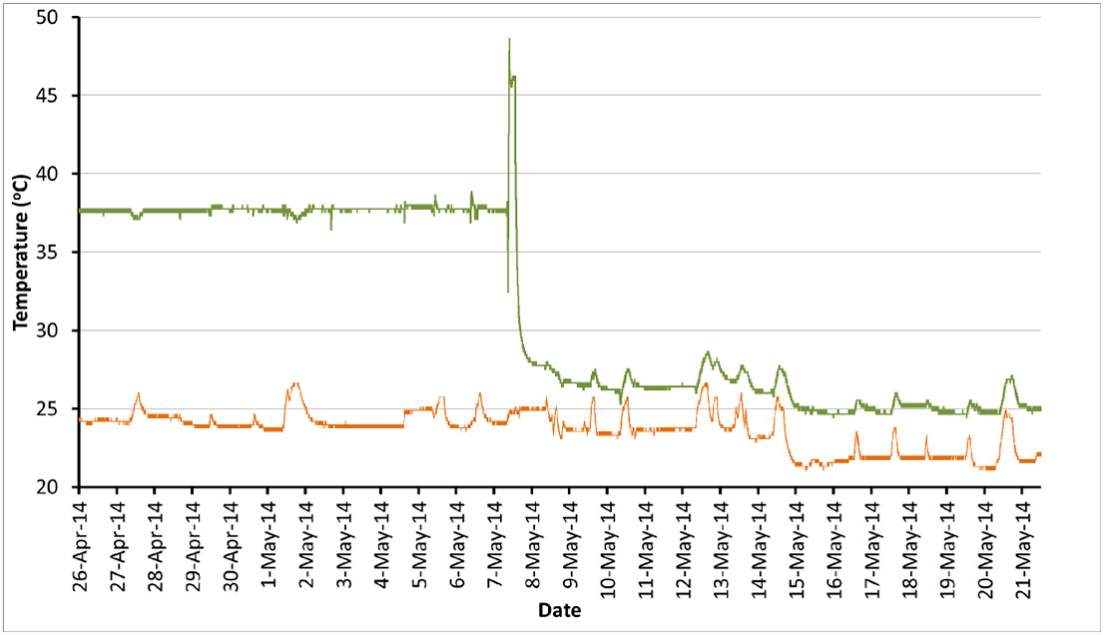
Concurrent temperature monitoring detects unwanted effects of room temperature on incubator internal temperatures. The monitoring system was using #1124 sensors to track temperatures in the room (orange line) and incubator (green line). In this case the incubator temperature sensor was located on the inner face of the main incubator door, outside the inner chamber glass doors.

Having established the capacity for short-term monitoring, we also investigated the potential for mining the larger volumes of data accumulated over time. After several months of monitoring, we examined the behavior of our tissue culture incubators, and made some important observations (Fig 4). Our laboratory conducts cell and tissue culture experiments using primary human cells and cell lines that require incubation at a range of temperatures. As shown here, an incubator was initially set to 37°C, and then later to 26°C. The peak preceding the transition is due to an elevated-temperature peroxide-sterilization cycle during incubator cleaning between cell batches. We noted that the tissue culture room temperature, already fairly warm, would intermittently rise further, which in turn resulted in elevated temperatures within the incubator when set to 26°C. Based on this data, we arranged with building services to reduce room temperature settings as far as possible (see decrease on May 14^th^), and when this proved insufficient to eliminate incubator temperature excursions, upgrades to the laboratory HVAC system were carried out.

**Fig 5:**
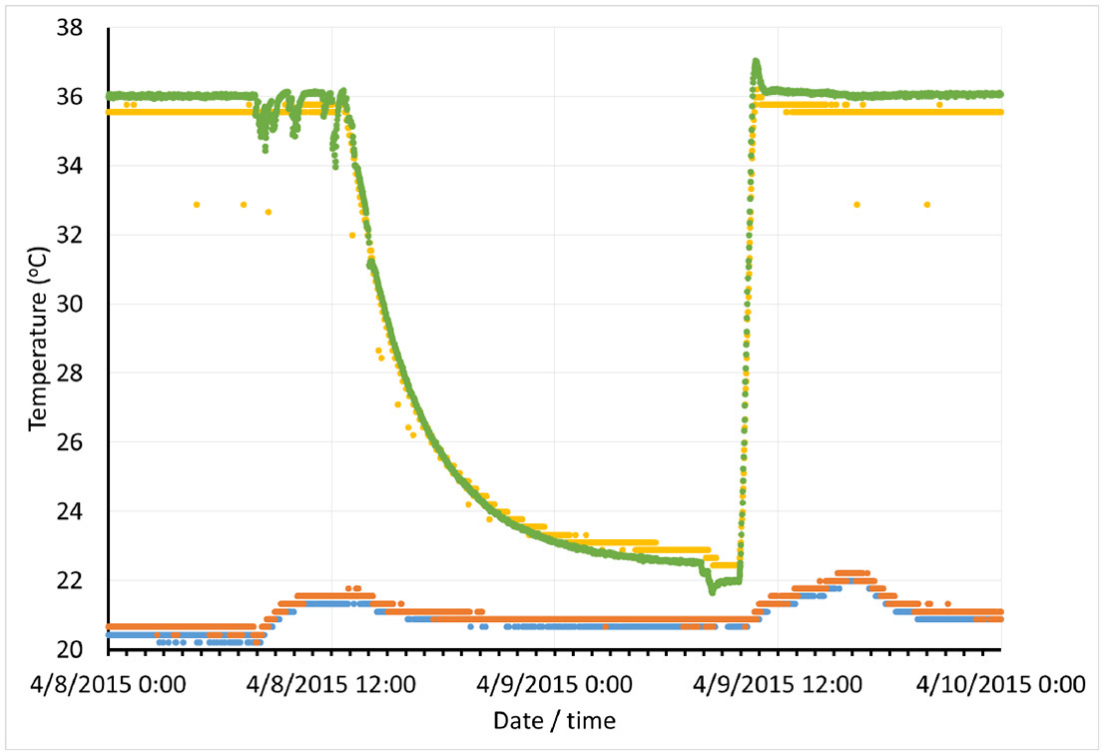
Sensor data documents equipment failure. Temperatures were monitored inside the incubator using both a #1124 temperature sensor (yellow line) and a thermocouple (green line) along with room temperature via two #1124 sensors (red and blue lines). The period of time immediately before, during and after a loss of temperature control could then be examined in greater detail.

In addition, long-term monitoring enabled us to capture rare failure events and analyse them after the fact in greater detail (Fig 5). One of our incubators suffered from intermittent, silent (no alarm, display shows normal status) and non-reproducible failures to maintain temperature. By continuously tracking temperature inside the incubator we were able to capture an event, providing the documentation necessary to obtain a warranty replacement of the incubator’s control electronics.

**Fig 6:**
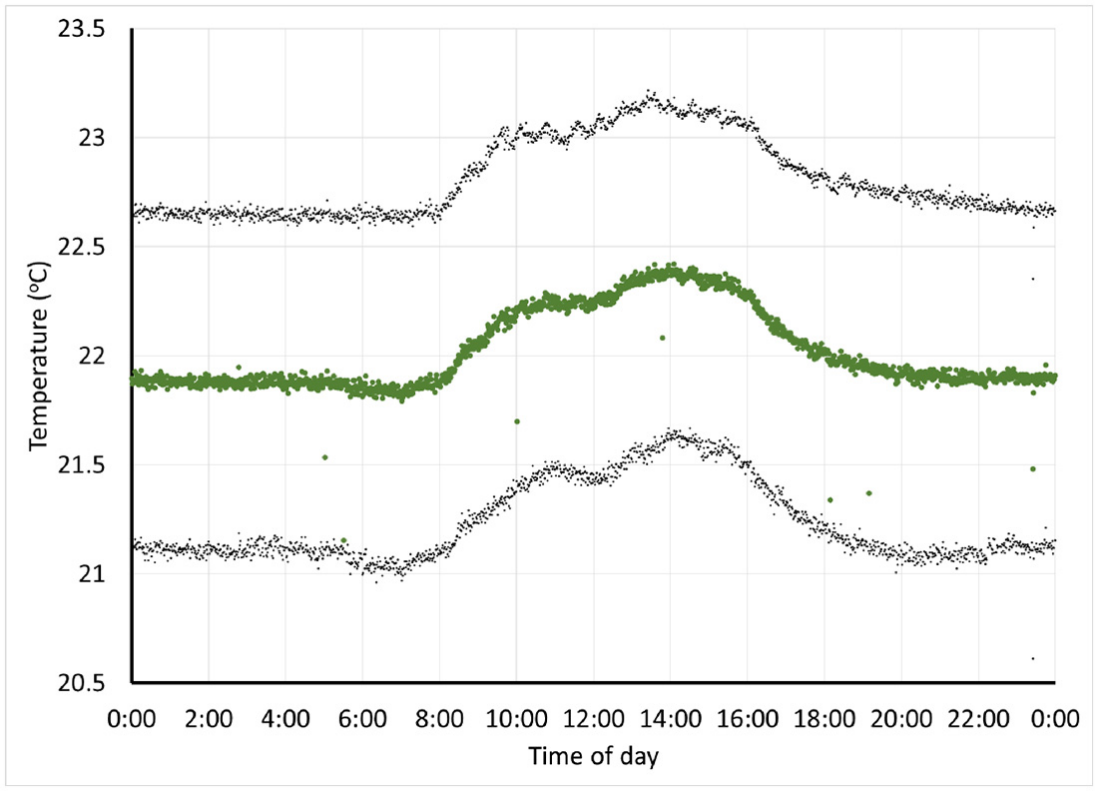
Daily tissue culture room temperature cycle. The green points represent the minute-by-minute average room temperature over the course of the day, obtained from >117,500 datapoints collected over the course of 104 days. Upper and lower grey points represent the average plus and minus one standard deviation.

**Fig 7:**
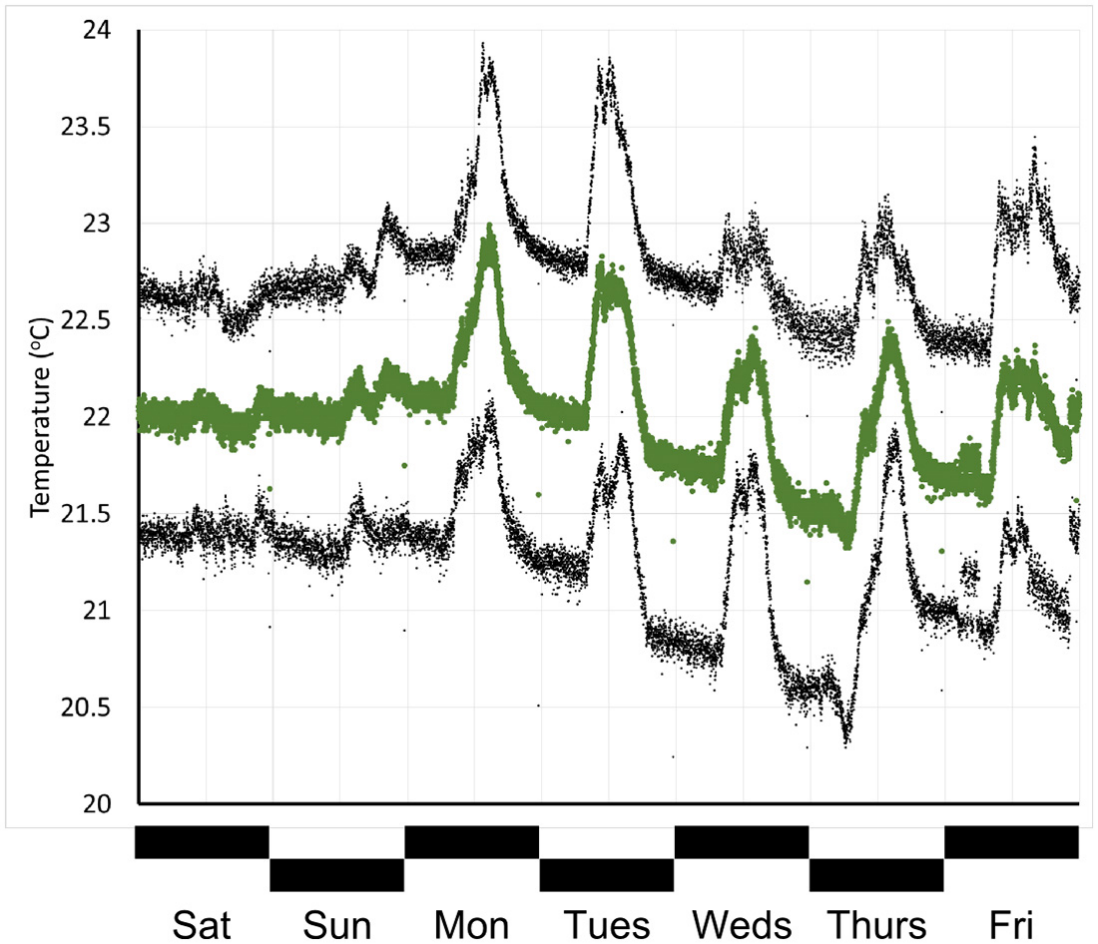
Weekly tissue culture room temperature cycle. The green points represent the minute-by-minute average room temperature over the course of the week, obtained from >117,500 datapoints collected over the course of 104 days. Upper and lower grey points represent the average plus and minus one standard deviation.

Once the HVAC system in our tissue culture space was upgraded, we were interested to observe the impact on room temperature. We assembled over 100,000 individual datapoints captured over the course of >100 days, and examined average daily (Fig 6) and weekly (Fig 7) temperature trends. At this depth, changes in temperature as the use level increases are clear, to the point that it is possible to identify a minor temperature drop around the lunch hour. Overall temperature decreased substantially (compare to Fig 4), providing confidence that the equipment was performing adequately to protect our cultured tissue samples.

Finally, as the sensors are powered from the Raspberry Pi via the USB interface, an adequate power supply is an important consideration. In particular, we initially tested the system using a 5V 1A power supply, and observed intermittent failures where the system would cease collecting data. Since transitioning to 5V 2A supply this issue has ceased. We also observed that an inline 9,000 mAh USB battery was able to sustain the system for on the order of one day after loss of external power, although this will vary depending on the precise combination of sensors attached.

## Conclusion

The monitoring system presented here permits access to important data about critical laboratory systems that are generally not captured outside of larger-scale industrial or clinical settings. By establishing an historical record, conditions can be retrospectively correlated with experimental outcomes for sensitive procedures such as primary cell culture and in vitro fertilization. The system can also be configured to warn the user via email when sensor readings move outside a preset range. As described the system can monitor temperature, pressure and circuit open / closed status, and as the software is open source it can easily be extended to additional commercially available sensors such as humidity, pH, motion, force, light, sound, charge, voltage, current, and RFID tag readers.

## Supporting Information

S1 Appendix Installation and setup instructions. (PDF)

S1 File Temperature sensor encapsulation mould. 3D printable STL files for a mould to encapsulate an 1124 temperature sensor inside a rubber stopper.
(ZIP)

S1 Table Components and suppliers. (PDF)
