# Supplementary material for: A Simple and Low-Cost Monitoring System to Investigate Environmental Conditions in a Biological Research Laboratory
Source: PLoS One. 2016 Jan 15;11(1):e0147140. doi: 10.1371/journal.pone.0147140 (PMC4714841; doi:10.1371/journal.pone.0147140)
Supplement: S1 Appendix — (PDF) [file pone.0147140.s001.pdf]

Instructions for setting up the monitoring system

Important considerations

Significant effort has been made to ensure the system performs as expected, however we are not able to test all combinations of conditions that might result in inaccurate readings, failure of internet connections etc. It is therefore very important to check system functioning on initial setup, and periodically thereafter. Ask whether the readings given by the sensors are realistic, whether they change as expected, and whether appropriate alerts are sent. For example, does a sensor in a refrigerator give expected readings? If not, check it against a thermometer placed in the same location. Are any deviations acceptable (do you need to know the temperature to a fraction of a degree, or is it enough to know when temperature rises a few degrees above normal)? If the sensor is removed from the refrigerator, do the readings begin to transition towards room temperature, and are alert emails sent as expected? In a multi-user environment, it may be preferable to have alert emails sent to a mailing list, rather than one individual, in case of absences, vacation etc. The software is released under the GPL V3.0 (see below) without any warranty, and is not certified or rated for critical applications in medical or clinical laboratory settings.

Initializing the Raspberry Pi

Two ways of setting up the system are described below. The scripted method involves starting your Raspberry Pi following the manufacturer’s instructions, and then running a script that will install the monitoring-system-specific software. If your RPi came with an SD card preloaded with NOOBS or the

Raspbian operating system, this method can be completed using only the RPi, temporarily connected to a keyboard and monitor. The .img file method requires several operations on another computer, but once the SD card is prepared it can be inserted into the RPi and the remaining configuration completed over your network. You should read through both methods before deciding which to use. Note that recent models of Raspberry Pi use a "micro" SD format, which may need an adapter for use in the SD card port of desktop or laptop computers.

Scripted RPI setup

If your Raspberry Pi does not come with an included SD card and the NOOBS (New Out Of the Box Software) operating system install manager, follow the instructions found on the Raspberry Pi web site to download and install it: http://www.raspberrypi.org/help/noobs-setup/

The instructions found on the website will address the formatting of an SD card. Follow them carefully or the SD card may not be formatted correctly.

When copying the files from the NOOBS folder to the SD card, ensure you copy only the items inside the folder and not the folder itself.

Starting up the RPi

Plug the RPi into a USB keyboard and monitor. Make sure the SD card, loaded with the NOOBS software, is inserted into the slot on the RPi.

Connect the power supply. The RPi does not have a power switch, so this is how it is turned on and off.

When the RPi is first being set up, a dialogue box will appear asking which operating system should be utilized. Choose the "Raspbian" operating system – at the time of this writing, the keystroke combination

that will trigger this is “<space bar>” (to select the default Rasbian operating system), “i” to start installation, and “<enter>” to confirm. Installation may take some time but will proceed automatically.

Once install is completed and the RPi rebooted, it should prompt for the username and password. By default, the username is “pi” and the password is “raspberry”. The characters of the password will not appear as they are typed; this is a security feature.

```
Debian GNU/Linux 7 raspberry ttyl


Raspberrypi login:
Password:
```

If your RPi boots directly to the Graphical User Interface (GUI, also known as the “desktop”), open a terminal window and run:

```
pi@raspberrypi:~$ sudo raspi-config
```

Under the “Boot Options” or “Enable Boot to Desktop / Scratch” menu option, set the computer to boot to the command line – then exit via the “Finish” option, reboot your RPi and log in as above.

The default password on the RPi should be changed for security reasons, or anyone who knows the IP address will be able to log in unless blocked by your firewall. Type the following command:

```
pi@raspberrypi:~$ passwd
```

The passwd command allows the password to be changed; a prompt will appear for the old password, then twice for the new password. Once this is done, the new password will be required for the next login. Again, the characters will not be seen as they are typed.

At this point, users familiar with the SSH secure shell network protocol may choose to access the RPi remotely. Once the RPi is connected to your network, see below under “Remote access” for instructions on determining the RPi’s IP address.

Connecting the 1124, 1115, 1140, 1141 phidget sensors and thermocouple

Connect the sensors to the RPi as follows:

i. Connect the RPi to the Internet via Ethernet cable or wifi adapter.

ii. Plug in the Phidget Interface Kit (PIK) to the RPi using a USB cord. If available, connect the appropriate sensors (1124 Temperature and/or 1115, 1140 and 1141 Pressure sensors) to the PIK analog ports using the sensor cable provided. Various digital sensors may also be connected to the PIK using the digital ports (see Phidgets documentation for details).

iii. If desired, connect a Phidget Temperature Sensor 4 Input to the RPi using a USB cord.

iv. Connect the thermocouple to the terminals on the Temperature Sensor 4 by connecting the red end of the thermocouple to a terminal associated with a number; the white end should be connected to the adjacent terminal associated with the letter ‘G’.

v. Boot (or reboot) the RPi

Installing Phidgets Compatibility Modules, Lighttpd and pChart Modules

In order for the RPi to use and interpret the scripts involving Phidget devices, the necessary Python libraries and drivers must be installed.

First, either log in to the RPi directly or via SSH. Download the script “setup.sh” from the server. This can be done by typing:

```
pi@raspberrypi ~ $ sudo wget
https://raw.githubusercontent.com/ungrinlab/monitor/master/setup.sh
```

Note that the above command should be all one line, despite having wrapped here to fit on the page. Now that the script has been downloaded, it can be executed by typing:

```
pi@raspberrypi ~ $ /bin/bash setup.sh
```

This will execute the script, which will download the necessary Phidget Libraries, phidget module, USB drivers, lighttpd server setup, and pChart modules. As the script is running, a prompt may appear requesting permission to install various components. If so, enter ‘Y’ to continue. You will also be asked for permission for the software to contact our server to log your install. This will only record your IP address, and if you choose not to allow this, enter ‘n’. Your choice here does not affect the functionality of your system. A message at the end of the script will appear saying that the installation is complete.

The RPi will now automatically run the script ‘monitoringsystem.py’ once every minute to obtain data from temperature, pressure, digital sensors, and the 3107 Thermocouple. To edit how often this script is triggered, users familiar with the linux crontab command may enter

```
pi@raspberrypi ~ $ sudo crontab -e
```

Presently the script is triggered once every minute, but longer intervals, more complex scheduling (only on certain days of the week, only during certain parts of the day, only when the RPi is rebooted / powered up, etc) are also possible. See the linux crontab documentation online for more information on how to use this functionality.

From this point on, configuration can be completed using the web interface.

Setup via .img file

Another way to set up the system is to download what is known as an “image file” – essentially a snapshot of an already-set-up monitoring system. This file is available as a .zip archive at:

https://github.com/ungrinlab/monitor/releases/download/v1.0/monitor_image.zip

On a computer with network access and an SD card port, follow the above link, and save the file somewhere you can find it – NB it is close to 2 GB in size. Then, extract the contents of the zip archive,

which should consist of a single large file entitled “monitor_image.img”. The precise steps for doing this will vary with your operating system, but commonly involve either right-clicking on it and selecting “Extract all” or double-clicking on it - in the latter case if the archive is opened but the .img file is not extracted, you may need to drag and drop or copy / paste it to a new location.

Once you have the .img file, you need to transfer it to an SD card of at least 8 GB capacity. On Windows, this may be accomplished using the Win32DiskImager software (navigate to http://sourceforge.net/projects/win32diskimager/ and click on the “Download” button). WARNING: Make sure you choose the correct drive (SD card) to write to, as data on the target drive will be lost – double check to be sure you have not selected your hard drive before pressing the “Write” button. Once this process is complete (this may take some time), transfer the SD card to the Raspberry Pi, and follow the hardware connection instructions listed above under “Connecting the 1124, 1115, 1140, 1141 phidget sensors and thermocouple”. After completing these steps and powering up the RPi, the remainder of the configuration can be completed using the web interface.

The default password should also be changed for security reasons, or anyone who knows the IP address will be able to log in unless blocked by your firewall. Use ssh for remote access as described below - the default username is “pi” and the password is “raspberry”. The characters of the password will not appear as they are typed; this is a security feature. Once logged in, type the following command:

```
pi@raspberrypi:~$ passwd
```

A prompt will appear for the old password, then twice for the new password. Once this is done, the new password will be required for the next login. Again, the characters will not be seen as they are typed.

As this setup method does not require the user to log in to the RPi, it assumes the user consents to logging this install. If you wish to disable this behaviour, perform the first boot of the RPi without an internet connection, log in and type:

```
pi@raspberrypi:~$ echo 1 > /home/pi/scripts/hasrun.txt
```

Remote access

In order to remotely access the RPi, it is necessary to first determine its IP address. There are three ways to do this. The first is to ask your network administrator to assign a “static” (constant) IP address to the RPi, and tell you what it is. This is particularly important if your network uses dynamic IP addressing and you find the RPi’s IP changing frequently.

The second is to observe the LED indicators on the RPi itself. A script has been included that will attempt to detect the IP address, and then use the LEDs to blink it out. The LEDs will flicker to indicate the beginning and end of the IP address. A series of short blinks will count up to the individual digits of the address, and a single long blink indicates a “.”. So for example, the IP address 136.159.176.175 would be rendered as the following blink pattern:

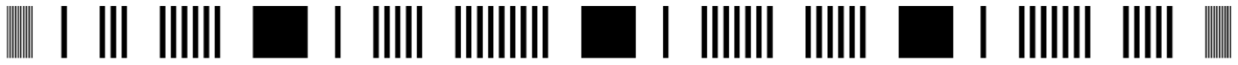

The third option requires that you be logged in to the RPi directly, where you can manually check the IP address. Type the following command:

```
pi@raspberrypi ~ $ sudo ifconfig
```

The command will list the network information for all Ethernet connections on the RPi. The wired Ethernet connection on the RPi corresponds to the eth0 network. The IP address should follow the words ‘inet addr:’(highlighted in the example below).

```
eth0      Link encap:Ethernet  HWaddr b8:27:eb:63:40:b8
          inet addr:172.28.145.190  Bcast:172.28.145.255  Mask:255.255.255.0
          UP BROADCAST RUNNING MULTICAST  MTU:1500  Metric:1
          RX packets:27224 errors:0 dropped:0 overruns:0 frame:0
          TX packets:733 errors:0 dropped:0 overruns:0 carrier:0
          collisions:0 txqueuelen:1000
          RX bytes:2801074 (2.6 MiB)  TX bytes:107019 (104.5 KiB)

lo        Link encap:Local Loopback
          inet addr:127.0.0.1  Mask:255.0.0.0
          UP LOOPBACK RUNNING  MTU:16436  Metric:1
          RX packets:0 errors:0 dropped:0 overruns:0 frame:0
          TX packets:0 errors:0 dropped:0 overruns:0 carrier:0
          collisions:0 txqueuelen:0
          RX bytes:0 (0.0 B)  TX bytes:0 (0.0 B)
```

To log out of the Raspberry Pi, type:

```
pi@raspberrypi:~$ logout
```

Accessing and Using the Web Interface

To access the web interface, direct your web browser to the RPi's IP address:

http://xxx.xxx.xxx.xxx

If you are unable to access this web page (or if you are only able to access it from devices directly connected to your network, but not from elsewhere), you may need to ask your network administrator to allow firewall access for http traffic to this address. You should see a screen similar to that shown in Figure 2, although the precise sensors displayed will depend on the configuration of your RPi. Note that until you have manually adjusted the settings, the configuration will not be accurate. The system does not have the

ability to automatically detect the sensors you have connected to it, so the sensor display region may be blank. To configure your system, click on the settings icon:

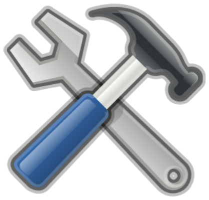

You will be asked for a username and password. By default, the username is “demo” and the password is blank. You should change these immediately, so that unauthorized users cannot change your settings. This may be done on the configuration page by typing new values into the “for sensor configuration” login and password fields and pressing the “Update settings” button.

On the configuration page, you can also specify the sensors that are connected, and the normal values you expect from them. Abnormal values will cause the indicators on the main page to change from green to red. The system will also send email alerts if set up to do so on this page as well. Note that at this time a Gmail account is required for the RPi to use to send emails from. It is recommended that this be a dedicated email account separate from the account you use for email correspondence, as the password is stored on the RPi. Once the email account and password are entered correctly, you can test this function by deliberately setting the “normal” range on a sensor so the current readings are outside them. When using Gmail to send emails it is necessary to allow the RPi to access the account. In order to do this login to google. Click on "My Account", followed by "Sign-in & security" and "connected apps and sites". Then turn on access for less secure apps.

Pressing the “Update settings” button will bring up a summary of the values stored, with a link to return to the main page.

From the main page you can also plot the data collected so far, by clicking on the chart icon:

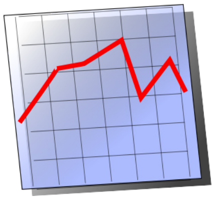

Below is an example of the pChart-generated graph:

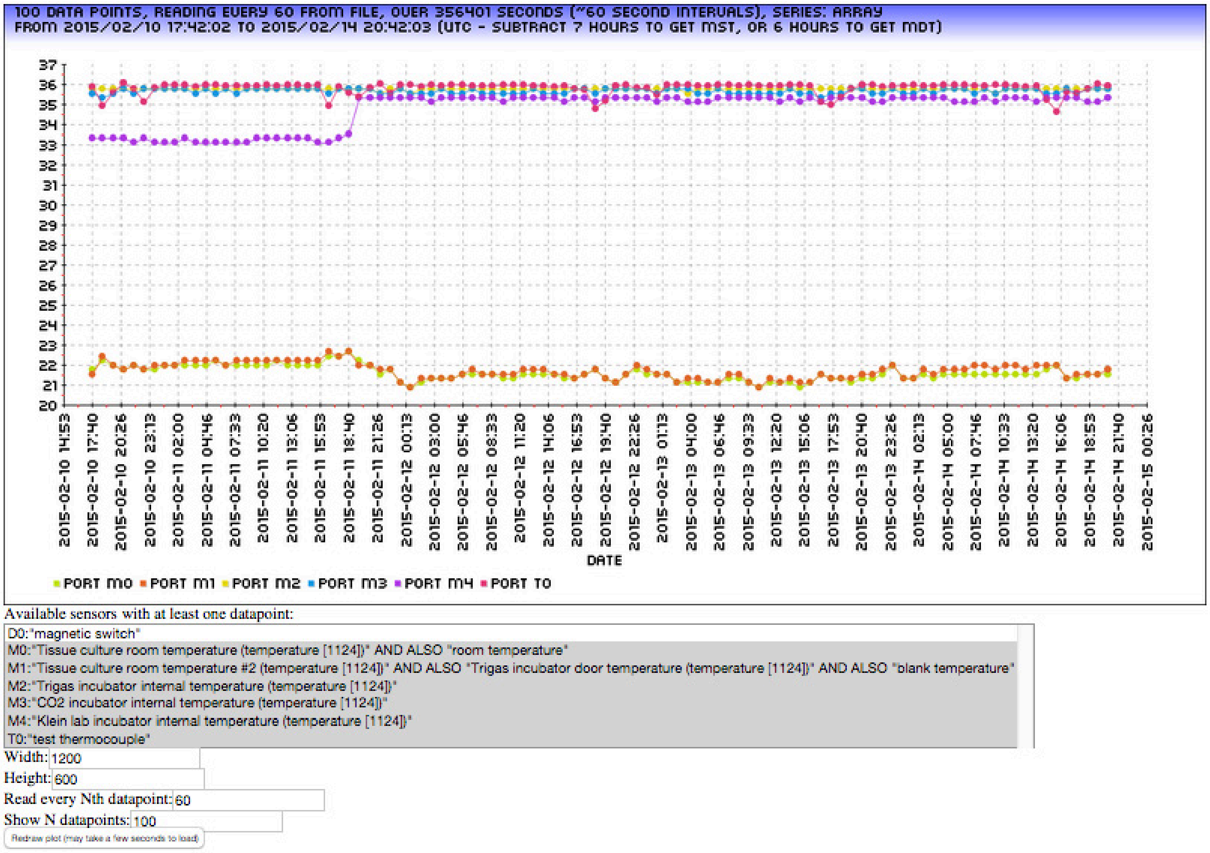

Supplementary Figure S1: Real-time plot of the monitoring systems data generated by pChart. Individual plots and the number of data points may be changed.

The log file created by the system can be accessed by clicking the download link on the main page:

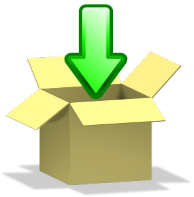

The page will load the entire log file containing all of the data points registered by all the sensors currently attached to the RPi. This may be copied and pasted into a spreadsheet for further analysis.

When the monitoring system detects a sensor that has exceeded its upper or lower limits as defined by the user, a separate recording of the instances are made in an alert log. This may be accessed by clicking on the alert log icon:

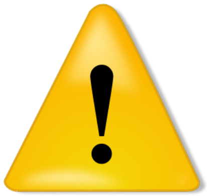

The alert log displays the readings for the particular sensor as well as whether an email alert has been sent.

3D printed mould for encapsulating a sensor inside a rubber stopper

Many pieces of equipment such as laboratory incubators are equipped with a port for the introduction of sensors, power supplies etc, often blocked with a standard rubber stopper. We designed a mould that would allow a Phidgets 1124 temperature sensor to be cast inside a modified rubber stopper. The STL file

for generating this mould on a 3D printer is included as S1_File.zip. The completed mould is shown here, along with the final product installed in an incubator:

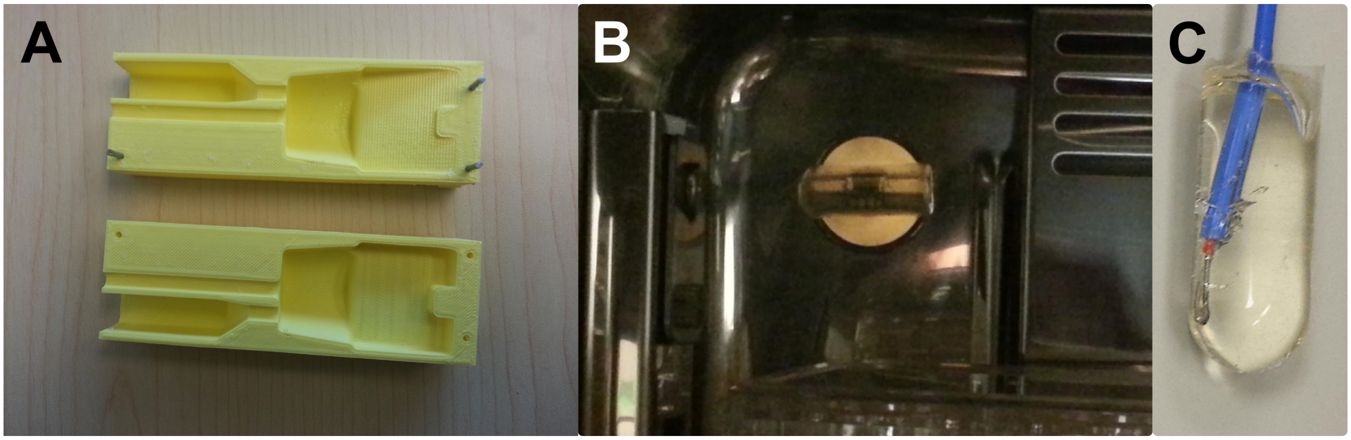

Supplementary Figure S2: Encapsulation of a temperature sensor in a rubber stopper. A two-part mould (**A**) was designed and 3D printed. A Phidgets 1124 temperature sensor was placed into it, and the mould filled with Sylgard 184 silicone rubber. The resulting stopper fits securely into a port at the back of a tissue culture incubator, allowing ethanol sterilization without damage to the embedded sensor (**B**). A simpler shape cast in a cryopreservation tube was also used to encapsulate a thermocouple sensor (**C**).

Customization and modification

The code written for this system is open source, and may be accessed either directly from the install web site (https://github.com/ungrinlab/monitor), or by following the installation instructions and editing it in place. On the RPi, the web interface files are found in the directory

```
/var/www
```

the install script is

```
/home/pi/setup.sh
```

and the sensor scanning script is

```
/home/pi/phidgets/monitoringsystem.py
```

The script to detect and blink out the IP address is

```
/home/pi/scripts/blink_IP.sh
```

The sensor interface used here is compatible with a wide range of additional sensors sold by Phidgets, including those for humidity, pH, load, light levels, RFID tags and current / voltage. Adapters are also available for industry-standard 4-20 mA sensors, which further broadens the range of detectors. Modifications to the software to support desired sensors are encouraged and would be relatively straightforward. The PhidgetInterfaceKit 8/8/8 also carries eight digital outputs, and controllers for stepper and servo motors are also available. USB WiFi connectors are available for the RPi, although we have not incorporated them into this system as setup is somewhat more complex than the simple Ethernet connection specified here. Guides are available online however and users with moderate computer skills should be able to connect their systems wirelessly in most instances. Solar power supplies, and converters suited to powering the system from deep-cycle RV / golf-cart batteries are available and would allow data logging in locations without mains power. We encourage users to take advantage of the "Comments" section of the online manuscript and the git repository to publicize and share code modifications and ideas, and collaborate on further improvements to make the system as broadly useful as possible.

The copyright statement for the software written to control this system is as follows:

©2015, Akshay Gurdita and Mark Ungrin (http://ucalgary.ca/ungrinlab). This program is free software: you can redistribute it and/or modify it under the terms of the GNU General Public License as published by the Free Software Foundation, either version 3 of the License, or (at your option) any later version. This program is distributed in the hope that it will be useful, but WITHOUT ANY WARRANTY; without even the implied warranty of MERCHANTABILITY or FITNESS FOR A PARTICULAR PURPOSE. See the GNU General Public License for more details.

This code is built upon code provided by Phidgets under the Gnu Lesser General Public License (LGPL V3.0) and the pChart PHP charting libraries, under the Gnu General Public License (GPL V3.0). Public-domain icons from the openclipart repository have also been used in several places.
