# Supplementary material for: A Simple and Low-Cost Monitoring System to Investigate Environmental Conditions in a Biological Research Laboratory
Source: PLoS One. 2016 Jan 15;11(1):e0147140. doi: 10.1371/journal.pone.0147140 (PMC4714841; doi:10.1371/journal.pone.0147140)
Supplement: S1 File — 3D printable STL files for a mould to encapsulate an 1124 temperature sensor inside a rubber stopper. (ZIP) [file pone.0147140.s002.zip › Instructions for 1124 encapsulation.pdf]

Instructions for encapsulating an 1124 temperature sensor inside a rubber stopper.

Two STL files are provided, representing the two halves of the mould. In our case they were printed on a MakerBot Replicator 2 printer in polylactic acid (PLA). One of these is diagrammed here:

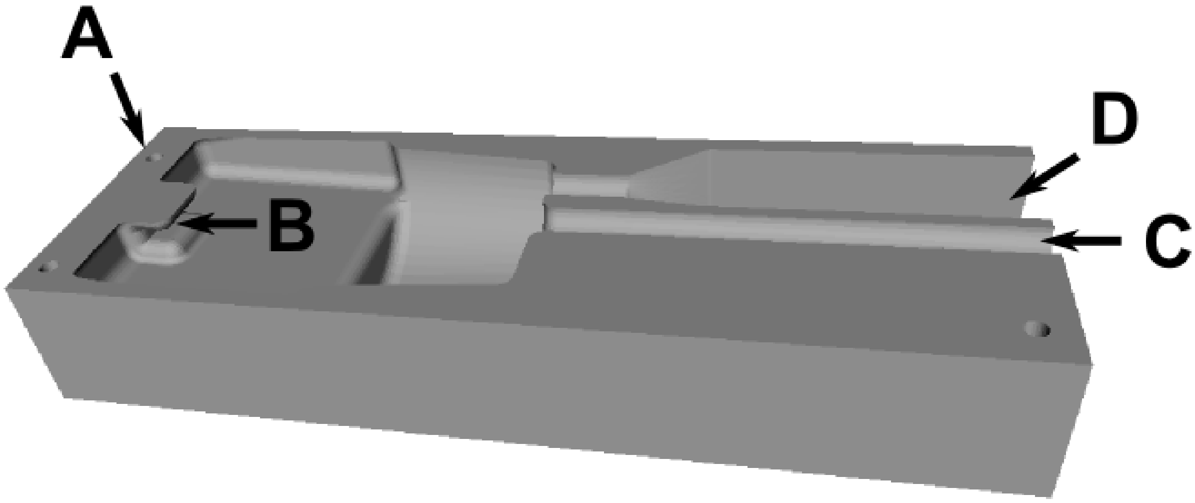

Holes are provided in the corners of the mould halves to accommodate optional pieces of 2 mm diameter wire to assist with alignment (**A**). After printing the two mould halves, the 1124 temperature sensor should be placed in the clip in Mould block 1 (**B**), with the wire connecting it to the interface kit connected and lying in the channel provided (**C**).

The two mould halves should then be assembled and aligned, and either clamped or taped in place. Some leakage should be anticipated so the assembly should be wrapped as tightly as possible in e.g. aluminum foil to capture it.

Silicone rubber pre-polymer (we used Dow Sylgard 184) should be prepared according to the manufacturer's instructions, and then be introduced through the funnel (**D**), taking care not to pour any into channel (**C**) as that channel must be open to allow air to escape from the mould. It is advisable to fill the mould at least part-way up the funnel to account for leakage during curing. After curing, the mould is disassembled, and the casting trimmed with a sharp knife to remove any excess material.
