# Supplementary material for: A Simple and Low-Cost Monitoring System to Investigate Environmental Conditions in a Biological Research Laboratory
Source: PLoS One. 2016 Jan 15;11(1):e0147140. doi: 10.1371/journal.pone.0147140 (PMC4714841; doi:10.1371/journal.pone.0147140)
Supplement: S1 Table — (PDF) [file pone.0147140.s003.pdf]

Item	Supplier	Catalog #	comments
Raspberry Pi 2 Model B	Newark element 14 (and many others)	38Y6469	required
Power supply, 5V 2A		68X2071	required - several alternatives exist, however as the Phidgets draw power through the RPi, we recommend at least a 2A capacity, as we have experienced erratic behaviour with 1A power supplies
Case for RPi		68X2068	optional, recommended to protect the RPi from damage
Patriot Fuel 9000mAh Tablet and Smartphone Mobile Rechargeable Battery	Amazon.ca	PCPB90002	optional, can be connected between the power supply and the RPi if power interruptions are a concern
PhidgetInterfaceKit 8/8/8	Phidgets.com	1018_2	required in order to connect 1124 temperature sensors, 1140 / 1141 pressure sensors and digital inputs
Precision temperature sensor		1124_0	optional, can connect up to 8 of these to the InterfaceKit, rated for temperatures of -30°C to +80°C
Absolute Air Pressure Sensor 20-400 kPa		1140_0	optional, can connect up to 8 of these to the InterfaceKit, rated for pressures of 20 kPa to 400 kPa
Absolute Air Pressure Sensor 15-115 kPa		1141_0	optional, can connect up to 8 of these to the InterfaceKit, rated for pressures of 15 kPa to 115 kPa
Sensor Cable 350cm		3004_0	optional, for extending the reach of the temperature and pressure sensors
Magnetic Contact Switch BR-1014		3560_0	optional, can connect up to 8 of these to the InterfaceKit digital inputs
PhidgetTemperatureSensor 4-Input		1048_0	required in order to connect 3107_0 Type K thermocouple
TPK-01 Bead Probe K-Type Thermocouple		3107_0	optional, can connect up to 4 of these to the PhidgetTemperatureSensor rated for temperatures of -50°C to +200°C. From our communications with technical support, the lower temperature limit is based on concerns of cracking the insulation if moved, but in a static situation where the probe will not be moved, it is suitable for much lower temperatures
K-Type Thermocouple Extension Wire		3112_0	optional, for extending the reach of the thermocouple probes
